# SMR/Theta Neurofeedback Training Improves Cognitive Performance and EEG Activity in Elderly With Mild Cognitive Impairment: A Pilot Study

**DOI:** 10.3389/fnagi.2020.00147

**Published:** 2020-06-16

**Authors:** Fabienne Marlats, Guillaume Bao, Sylvain Chevallier, Marouane Boubaya, Leila Djabelkhir-Jemmi, Ya-Huei Wu, Hermine Lenoir, Anne-Sophie Rigaud, Eric Azabou

**Affiliations:** ^1^Department of Clinical Gerontology, Broca Hôspital, Assistance Publique-Hôpitaux de Paris (AP-HP), Research TEAM EA4468, Paris Descartes University, Paris, France; ^2^Clinical Neurophysiology Laboratory, Department of Physiology, Raymond Poincaré Hôspital, Assistance Publique-Hôpitaux de Paris (AP-HP), INSERM U1173, University of Versailles Saint Quentin en Yvelines, Garches, France; ^3^Versailles Engineering Systems Laboratory (LISV), University of Versailles Saint Quentin en Yvelines (UVSQ), Vélizy, France; ^4^Clinical Research Unit, Avicenne Hospital, Assistance Publique-Hôpitaux de Paris (AP-HP), Bobigny, France

**Keywords:** neurofeedback, electroencephalography, elderly, Alzheimer, mild cognitive impairment

## Abstract

**Background**: Neurofeedback (NF) training, as a method of self-regulation of brain activity, may be beneficial in elderly patients with mild cognitive impairment (MCI). In this pilot study, we investigated whether a sensorimotor (SMR)/theta NF training could improve cognitive performance and brain electrical activity in elderly patients with MCI.

**Methods**: Twenty elderly patients with MCI were assigned to 20 consecutive sessions of sensorimotor (SMR)/theta NF training, during 10 weeks, on a basis of two sessions each week. Neuropsychological assessments and questionnaires, as well as electroencephalogram (EEG), were performed and compared between baseline (T0), after the last NF training session at 10 weeks (T1), and 1-month follow-up (T2).

**Results**: Repeated measures ANOVA revealed that from baseline to post-intervention, participants showed significant improvement in the Montreal cognitive assessment (MoCa, *F* = 4.78; *p* = 0.012), the delayed recall of the Rey auditory verbal learning test (RAVLT, *F* = 3.675; *p* = 0.032), the Forward digit span (*F* = 13.82; *p* < 0.0001), the Anxiety Goldberg Scale (*F* = 4.54; *p* = 0.015), the Wechsler Adult Intelligence Score–Fourth Edition (WAIS-IV; *F* = 24.75; *p* < 0.0001), and the Mac Nair score (*F* = 4.47; *p* = 0.016). EEG theta power (*F* = 4.44; *p* = 0.016) and alpha power (*F* = 3.84; *p* = 0.027) during eyes-closed resting-state significantly increased after the NF training and showed sustained improvement at a 1-month follow-up.

**Conclusion**: Our results suggest that NF training could be effective to reduce cognitive deficits in elderly patients with MCI and improve their EEG activity. If these findings are confirmed by randomized controlled studies with larger samples of patients, NF could be seen as a useful non-invasive, non-pharmacological tool for preventing further decline, rehabilitation of cognitive function in the elderly.

**Clinical Trial Registration:** This pilot study was a preliminary step before the trial registered in www.ClinicalTrials.gov, under the number of NCT03526692.

## Introduction

Mild cognitive impairment (MCI) is defined as a symptomatic phase between normal cognitive functioning and mild dementia characterized by cognitive changes with preserved social and occupational functioning (Albert et al., 2011). MCI may refer to the earliest manifestation of Alzheimer’s disease or a neurodegenerative disease with progressive deterioration in cognitive function and daily function. Managing MCI to prevent dementia is crucial. Currently available interventions targeting cognitive rehabilitation in elderly patients with cognitive impairment are very limited. Increasing efforts are being made to develop pharmacological and non-pharmacological interventions to stop or prevent cognitive decline and dementia in the elderly. Neurofeedback (NF) is one promising technique in this field (Arani et al., 2010; Askovic et al., 2017; Aggensteiner et al., 2019). NF uses real-time displays of brain activity, most commonly electroencephalography (EEG), to promote self-regulation of brain function. This technique, defined as a closed-loop application, helps individuals to control or modify their cortical activity through learned self-regulation. The aims are to obtain better alertness, lower anxiety, and consequently improve attention, memory, and better behavior. Several well-conducted studies have shown the effectiveness of NF in cognitive functions (Arns et al., 2014; Gevensleben et al., 2014) particularly in the field of attention-deficit/hyperactivity disorder (ADHD; Micoulaud-Franchi et al., 2015) epilepsy (Egner and Sterman, 2006), autism (Coben et al., 2010), depression (Micoulaud-Franchi et al., 2015), and anxiety (Hammond, 2005). This last decade, knowledge regarding principles and technical aspects to achieve the efficient use of EEG NF improved a lot (Marzbani et al., 2016; Arns et al., 2017; Enriquez-Geppert et al., 2017). Among the NF protocols, the NF sensorimotor (SMR NF) appears to be one of the most effective (Dessy et al., 2020). It is based on brain oscillations of 12–15 Hz frequency training, recorded over central scalp regions, and generated in a reticular-thalamocortical network (Sterman, 1996, 2000). It was suggested that SMR NF training might facilitate thalamic inhibitory mechanisms and block motor activity that interferes with information processing (Sterman, 1996). SMR NF training improved declarative memory (Hoedlmoser et al., 2008; Gruzelier, 2014; Schabus et al., 2014; Kober et al., 2015; Cho et al., 2016; Reichert et al., 2016) and attention (Vernon et al., 2003; Egner and Gruzelier, 2004; Lecomte and Juhel, 2011; Kober et al., 2015) in elderly patients. Theta rhythm (4–7 Hz) is associated with neurological and psychological functions in the limbic system. It is linked with the control of arousal, affective and mental states (Ros et al., 2009). A randomized controlled study of healthy older adults showed a reduced theta absolute power in EEG and improved verbal comprehension index and revised Weschler adult intelligent score (WAIS-revised) after 30 training sessions of NF (Becerra et al., 2012). In another randomized placebo-controlled study, the experimental group of healthy older adults showed an improvement in working memory performance and activation power in theta and beta frequency bands at frontal and alpha at temporal regions after the SMR NF training (Campos da Paz et al., 2018). Patients with MCI trained with alpha NF protocols showed an increment in alpha activity and improvement in memory performance (Lavy et al., 2019). We hypothesized that NF training might be useful in elderly patients with MCI. In this study, we investigate the effects of a SMR/theta NF training protocol on cognitive performances, psycho-affective scores and EEG activities in elderly patients with MCI. The goal of this pilot study was beyond understanding the physiological mechanisms of the effects of the NF technique. Our objective rather is to assess whether NF could be effective in improving cognitive decline in the clinical specific population of elderly patients with MCI.

## Materials and Methods

### Study Population

Thirty-three right-handed patients from both sex, aged between 65 and 90 years, with an education level of at least 9 years of school, were recruited from the memory center of the geriatric department of Broca University Hospital in Paris. The inclusion criteria were: (a) subjective memory complaint confirmed by an informant; (b) a Mini-Mental Status Examination (MMSE) score >20 (Folstein et al., 1975); (c) MCI criteria according to Petersen (2004); (d) performance at/or below 1.5 standard deviations from the mean for age and education-matched norms on more than one of the neuropsychological tests; (e) the ability to provide written informed consent; (f) preserved activity of daily living; and (g) absence of dementia. Exclusion criteria were elderly persons under guardianship, resident in nursing facilities, participating in other trials, psychotropic medication, and medical history of neurological or psychiatric disease such as epilepsy, brain tumor, head trauma, stroke, bipolar disorder, schizophrenia, alcoholism. Before inclusion, all participants signed an informed consent form after they were explained verbally and in a written document about intervention, assessment conditions.

### Design and Procedure

This pilot study was conducted from March 2017 to April 2018. It was conducted following the CONSORT statement for non-pharmacological treatment interventions. The study protocol was approved by the ethics committee of the Paris Descartes University Institutional Review Board (reference code: 2017-12; 28/02/2017). Cognitive and psycho-affective scales were assessed for each patient by a trained senior neuropsychologist. Alternative test versions or forms were used during post-NF training when possible to avoid learning effects due to repeated tests. For each patient, EEG was recorded by a trained EEG technician, and EEG data were analyzed by a senior neurophysiologist.

### The NF Training Procedure

The NF sessions were conducted at the “La Collegiale gerontological Hospital” in Paris. Before starting the NF training program, each participant receives a demonstration of the NF EEG technique with explanations of its principle and its non-invasiveness.

EEG signals for SMR/theta training were recorded from the centro-median channel Cz according to the International 10-20 system and the instructions for the SMR NF protocol (Campos da Paz et al., 2018). An EEG Digitrack Biofeedback plus module, Inc Elmiko Medical device was used for the NF training. SMR was stimulated while theta waves (4–8 Hz) and beta waves (21–30 Hz) were suppressed. The ground electrode was set at the right mastoid and the reference at the left mastoid. All impedances were maintained below 5 KOhms (kΩ). The bandpass filtered between 0.1 and 60 Hz with a notch filter at 50 Hz to eliminate signal electrical interference. EEG signal was inspected and analyzed for all kinds of artifacts such as movements, coughs, jaw contraction, eye blinks. The sampling frequency was 512 Hz. Parameters used in this study such as the number of sessions, the duration of the training and the choice of the tasks were designed following some previous works methods (Marzbani et al., 2016; Arns et al., 2017; Enriquez-Geppert et al., 2017), current guidelines (Dessy et al., 2020; Ros et al., 2020; Steingrimsson et al., 2020) and experts recommendations for EEG NF training implementation (Micoulaud-Franchi et al., 2015; Zivoder et al., 2015; Banerjee and Argaez, 2017). The NF training was carried out for 20 sessions, twice a week for 10 weeks. Each session lasted 1 h 15 min that included 15 min of preparation and installation of the electrodes, verification of the impedance and adjustment of the thresholds, 45 min of training EEG NF, and 15 min of debriefing. Following a previous study on the acceptability of EEG NF in elderly people that our research team carried out at the Broca University Hospital in Paris, we selected twelve tasks from the listed games of the EEG Digitrack System (EEGDigitrack Biofeedback plus module, Inc Elmiko Medical) who received the most compliance. Six were displayed at the first 10 sessions and six at the last 10 sessions. Each task ran for 3 min corresponding to a new game, the last one was a video of a historical reportage watched for the last 20 minutes, divided into four rounds of 5 min with 20 s break between. The end of the movie was placed at the beginning of the next training session. The order of tasks was identical across the session and patient. The subjects received visual and auditory feedback through animated graphics shown on a screen. To modulate brain activity in the selected frequency bands, bad contrast and a particular sound were indicated each time the brainwaves were not reached. As second feedback, horizontal bars were presented on the corner of the patient’s screen showing his/her real-time SMR power, theta power, upper beta power according to the predefined threshold. When the expected band power reached the threshold, the color of the bar changed from red to green. Participants were instructed to try to voluntarily increase these bars to the green color. The target values were based on measurements during the first 3 min of each training session. Average thresholds were automatically calculated for each participant and each session, to 50% of the maximum amplitude. However, the professional could adjust them manually during the training session if a patient was encountering severe difficulties to reach them and if his motivation was dropping. NF training sessions were performed by a doctoral student trained to EEG and EEG NF application (FM).

### Neuropsychological Assessments

Neuropsychological tests assessed attention, working memory, executive functions, episodic memory, and global cognitive functions (Table 1). The mood was assessed by the Geriatric Depression Scale (GDS), a brief 30-item questionnaire to monitor depression over time in clinical settings (Yesavage et al., 1982). Anxiety was assessed with the Goldberg Anxiety Scale, the most commonly used rating scale to measure the severity of perceived anxiety symptoms (Hamilton, 1959). Quality of life was assessed with the Quality of Life Scale for Older French People, illustrating various dimensions such as daily and social activities in the environment, social and familial relationships, physical and functional health and mental health (Echelle de Qualité de Vie adaptée aux Personnes Agées; EQVPA; Petit et al., 2014). Subjective memory complaints were assessed with the French versions of the Mac Nair Cognitive Difficulties Scale measuring subjective difficulties in attention, memory, perception, and psychomotor abilities (Poitrenaud et al., 1997).

**Table 1:** List of neuropsychological tests administered before, after-NF training and at follow-up.

Neuropsychological tests	Cognitive function assessed	Description
Global assessment of cognitive functions:
MoCa (Version 2; range from 0–30)	Visuospatial/executive	Score between 21 and 25 = MCI
	Naming	
	Memory	
	Attention	
	Language	
	Abstraction	
	Orientation	
MMSE (Range from 0–30)	Brief quantitative cognitive measures	Score between 23 and 26/30 = MCI
	Orientation	
	Registration	
	Attention	
	Calculation	
	Recall	
	Language	
**Episodic memory**:		
RAVLT (4 lists of 15 words)	Declarative memory	A 15 noun word list, Ability to encode, Combine, store, and recover verbal information.
Story recall (A or B)	Declarative memory	Two immediate recalls and a delayed recall (30 min)
Rey-Osterrieth Complex Figure copy score (or Taylor Complex Figure)	planning, visuospatial processing Visual declarative memory	Copy completion time, 30 min recall score.
**Working memory and executive functions**:		
TMTA (Trail Making Test part A)	Sustained attention	Capacity to maintain and attentional activity over some time
TMTB (Trail Making Test part B)	Alternating attention	Capacity to shift in focus and tasks (B/A time)
K.T	Executive function	Capacity to discriminate stimuli
Forward and Backward Digit Span (Weschler Adult Intelligent Scale 4th edition)	Working memory	Capacity to process speed and attention control.
**Instrumental functions**:		
Semantic and lexical	Fluency language Semantic memory	In 2 min

### EEG Data Acquisition

For each participant, EEG was recorded and the EEG power spectrum was calculated in pre (T0) and post NF training at T1 and T2 using the Fourier transform. An electrocap of 19 scalp locations (Electro Cap, International Inc. Elmiko Medical) according to the International 10–20 EEG placement system, in Fp1, Fp2, F7, F3, Fz, F4, F8, T3, C3, Cz, C4, T4, T5, P3, Pz, P4, T6, O1 and O2 with reference at the left mastoid and the ground at the right mastoid was used. The recording used the sampling frequency of 500 Hz and a band-pass filter (0.01–100 Hz). Multichannel amplifier (EEGDigitrack SimplEEG32, Inc. Elmiko Medical sp.z.o.o Limited) was used. Impedance was kept below 5 KΩ for EEG. EEG artifacts were automatically rejected. Then, two baseline conditions were recorded including the eyes-open baseline (EOB) condition and an eyes-closed baseline (ECB) condition. Each condition was lasted for 3 min and was repeated three times (18 min recording in total). During the ECB condition, participants were asked to close their eyes but stayed awake. The power of the EEG rhythms, such as delta (0.5–3.9 Hz), theta (4–7 Hz), alpha (8–12 Hz), SMR (12–15 Hz), and lower beta (13–20 Hz) were computed from the moving averaged EEG power spectra.

### EEG Data Analysis

We have removed the artifact using independent component analysis (ICA) decomposition (Gramfort et al., 2014), removing independent components linked with ocular movements and muscular artifacts. The recorded EEG also passed a rejection test with a covariance-based approach (Barthelemy et al., 2019), ensuring that there are no remaining artifacts. All the ECB conditions were concatenated to estimate the variation in the frequency domain, relying on the Welch method. Using the fast Fourier transform, the power spectral density of the EEG signals (PSD) was calculated to extract the relative power for each frequency band and was estimated as a log-ratio for a reference period using MNE in Python 3.6 (Gramfort et al., 2014). The PSD for frequency *f* at time *t* is estimated as:

(1) $$PSD(t,f)=\log\left(\frac{A(t,f)}{\bar{A}}\right)$$

with the average power spectrum *Ā* estimated as

(2) $$A(t,f)=\frac{1}{N}\sum_{i=1}^{N}\text{Welch}({\text{X}}_{i},t-\Delta t,t)$$

where *X*_i_ is the EEG signal for electrode *i*. The reference power spectrum *Ā* is defined as

(3) $$\bar{A}=\sum_{t=t_{0}}^{t_{1}}\sum_{i=1}^{N}\text{Welch}(X_{i},t-\Delta t,t)$$

Here, the reference periods are the 5-s spans before the condition starts. *t*_0_ and *t*_1_ (in the 3rd equation) denote the start and end of the baseline, here *t*_0_ = −5 s and *t*_1_ = 0 s.

### Statistical Analysis

All statistical analyses were carried out using R statistical software version 3.3.2 (R Foundation for Statistical Computing, Vienna, Austria). Descriptive statistics were used to describe the demographic and clinical characteristics of the participants and other variables of the study. Neuropsychological and psycho-affective data were summarized in the form of mean and deviation standard (SD) for quantitative variables, while computed PSD data were in the form of mean and standard error mean (SEM). The resulting spectrogram is an image of the topographic distribution of the EEG to estimate the frequencies in the non-stationary signal by introducing a time dimension in the frequency analysis and compare spectrums at Baseline, post-NF training, and 1-month follow-up. Log-ratio of the PSD varies with time and different colors on the image represent different EEG values. Since the parametric conditions were confirmed, the repeated measures ANOVA test was used to assess the effects of the NF training on the neuropsychological, psycho-affective variables, and PSD values between the three endpoints times (T0, T1, and T2). *Post hoc* analysis was performed using paired Student *t*-tests. A *p*-value below 0.05 was considered significant.

## Results

**Figure 1:**
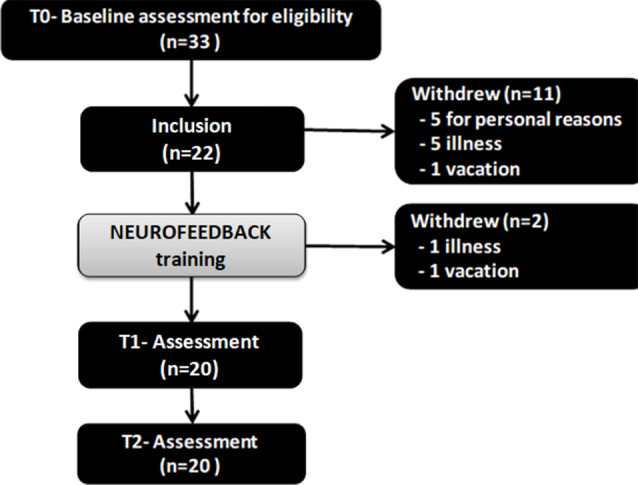
The flow chart of the study. Twenty-two patients were included and underwent baseline evaluation T0, but two of them left the study after the T0 evaluation and before T1 and T2 assessments: one for vacations and one due to disease. Only 20 patients completed the entire neurofeedback (NF) training sessions, the post-intervention assessment T1, and follow up assessment T2. Notes: T0 = baseline; T1 = Post-intervention; T2 = 1-month follow-up.

From the 33 patients enrolled, 11 dropped out (did not turn up for sessions after the baseline assessment or did not complete up the sessions). Twenty-two patients were included and underwent baseline evaluation T0, but two of them left the study after the T0 evaluation and before T1 and T2 assessments: one for vacations and one due to disease. Only 20 patients completed the entire NF training sessions, the post-intervention assessment T1, and follow up assessment T2. Figure 1 represents the flow chart of the study. The mean age of the 22 initially included participants was 76.1 years (SD 5.9), there were more women than men (77.3% of women), the mean education level was high (14. 9 ± 2.6). These details are summarized in Table 2.

**Table 2:** Socio-demographic characteristics of the 22 initially included patients.

Variable	Total (*n* = 22)
Age (years), Mean (SD)	76.1 (5.9)
Women, % (*n*)	77.3% (17)
MMSE Score, Mean (SD)	25.4 (2.8)
MoCa Score, Mean (SD)	23.1 (2.5)
Education, years, Mean (SD)	14.9 (2.6)

*Abbreviations: *MoCa* = Montreal Cognitive Assessment and orientation (Nasreddine et al., 2005; Nasreddine and Patel, 2016), *MMSE* = Mini Mental State Examination (Folstein et al., 1975)*.

### Effects of the NF Training on Cognitive and Psycho-affective Items

**Figure 2:**
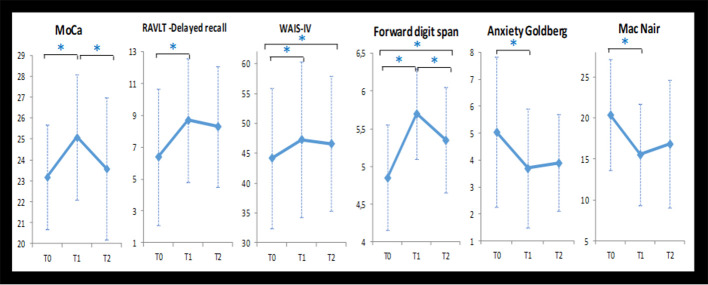
Neuropsychological and cognitive items with significant score change after the NF training for the 20 elderly patients: baseline (T0), post-intervention (T1), and 1-month follow-up (T2). Note: *statistical significant difference (*p* < 0.05).

Changes in scores for cognitive and psycho-affective measures after the NF training at T1 (post-intervention, and at 1 month follow up (T2) are presented in Table 3 as well as their baseline values at T0. Repeated ANOVA measures reveal that from baseline to post-intervention (T0, T1, and T2), participants showed significant improvement in the MoCa (*F* = 4.78; *p* = 0.012), the delayed recall of the RAVLT (*F* = 3.675; *p* = 0.032), the Forward digit span (*F* = 13.82; *p* < 0.0001), the Anxiety Goldberg Scale (*F* = 4.54; *p* = 0.015), the Wechsler Adult Intelligence Score–Fourth Edition (WAIS-IV; *F* = 24.75; *p* < 0.0001), and the Mac Nair score (*F* = 4.47; *p* = 0.016). *Post hoc* analysis student paired *t*-test found significant improvement for each of these items between the baseline (T0) and T1 (*p* < 0, 05). These beneficial effects of the NF training sustained at 1-month follow-up (T2) for most of the items except the MoCa and the Forward digit span. Figure 2 features neuropsychological and cognitive items with significant score change after the NF training for the 20 elderly patients: baseline (T0), post-intervention (T1), and 1-month follow-up (T2).

**Table 3:** Scores changes on cognitive and psycho-affective measures from baseline (T0) to post-intervention (T1) and 1-month follow-up (T2).

Variables	T0	T1	T2				T1–T0	T2–T0	T2–T1
	(*n* = 20)	(*n* = 20)	(*n* = 20)	Repeated measures ANOVA			Student’s *t*-test		
	mean ± SD	mean ± SD	mean ± SD	*F*_(2,38)_	*p*-values	Cohen’s *f*	*p*-values		
MMSE	25.3 ± 2.8	25.9 ± 2.5	25.5 ± 3.5	0.474	0.625	0.035			
MoCa	23.2 ± 2.5	25.1 ± 3	23.6 ± 3.4	4.78	0.012*	**0.008**	0.007*	0.937	0.015*
Logic memory-trial1	9.5 ± 4.6	10.125 ± 4.6	9.575 ± 4.4	0.293	0.747	0.057			
Logic memory-trial2	13.25 ± 4.6	14.425 ± 4.4	13.2 ± 4.5	1.652	0.201	0.028			
Logic memory-recall	11.325 ± 5.2	12.625 ± 4.7	12 ± 5.2	0.976	0.383	0.052			
RAVLT-Total	41.4 ± 10.7	41.9 ± 11.2	39.9 ± 15.1	1.866	0.164	0.028			
RAVLT-Delayed recall	6.4 ± 4.3	8.7 ± 3.9	8.3 ± 3.8	3.675	0.032*	**0.113**	0.027*	0.079	0.655
TMT-A	46.85 ± 16.7	45.25 ± 15.01	52.8 ± 21.4	2.908	0.063	>0.001			
TMT-B	173.35 ± 121	144.3 ± 95.7	161.3 ± 122.5	0.990	0.378	0.001			
REY—copy	28.25 ± 5.9	28.675 ± 7.5	29.525 ± 4.6	0.412	0.664	0.012			
REY—completion time	209.75 ± 79.6	187.9 ± 73.8	190.7 ± 64.5	1.041	0.360	0.036			
REY—recall	10.9 ± 5.7	10.925 ± 6.9	12.325 ± 7.4	1.442	0.245	0.120			
WAIS-IV	44.2 ± 11.8	47.3 ± 13.1	46.6 ± 11.3	24.753	<0.0001*	**0.009**	0.976	<0.0001*	<0.0001*
Forward digit span	4.85 ± 0.7	5.7 ± 0.6	5.35 ± 0.7	13.824	<0.0001*	**0.003**	<0.0001*	0.004*	0.049*
Backward digit span	3.6 ± 0.6	3.95 ± 0.7	3.8 ± 1	1.744	0.184	0.058			
Phonetic verbal fluency	18.3 ± 5.4	19.7 ± 6.7	17.8 ± 6.9	0.972	0.385	0.050			
Categorical verbal fluency	19.1 ± 5.6	22.95 ± 7	19.1 ± 7.6	2.860	0.066	0.038			
Anxiety Goldberg	5.05 ± 2.8	3.7 ± 2.2	3.9 ± 1.8	4.545	0.015*	**0.103**	0.007*	0.06	0.635
Depression GDS	8.11 ± 4.3	7.35 ± 4	8.65 ± 3.6	0.778	0.352	>0.001			
Mac Nair	20.4 ± 6.8	15.55 ± 6.2	16.85 ± 7.8	4.469	0.016*	**0.014**	0.012*	0.095	0.267
EQPVA	9.25 ± 2.3	10.15 ± 2.3	10.25 ± 2.2	2.491	0.092	0.014			
KT—Correct answers	33.1 ± 10.4	34.25 ± 10.7	33.7 ± 11	0.488	0.616	0.002			
KT—Omissions	5.05 ± 3.3	4.15 ± 3.5	4.25 ± 2.7	0.770	0.468	0.037			
KT—Mistakes	0.9 ± 0.9	0.55 ± 0.8	0.9 ± 1.5	1.120	0.333	0.037			

Note: *MoCa* = Montreal Cognitive Assessment and orientation (Nasreddine et al., 2005; Nasreddine and Patel, 2016), *MMSE* = Mini-Mental State Examination (Folstein et al., 1975), *RAVLT* = Rey Auditory Verbal Learning Test (Ricci et al., 2012), *TMTA&B* = Trail Making Test part A and part B (Reitan, 1955; Salthouse, 2011), *K.T* Based cancellation Test (Wu et al., 2016). Rey Figure (Rey, 1941; Osterrieth, 1944), Story recall (Barbizet et al., 1965). Wechsler Adult Intelligence Scale—Fourth Edition (WAIS-IV), San Antonio, TX: Pearson, 2008). *Significance at *p* < 0.05.

### Effects of NF Training on EEG’s Bands’ Powers

**Figure 3:**
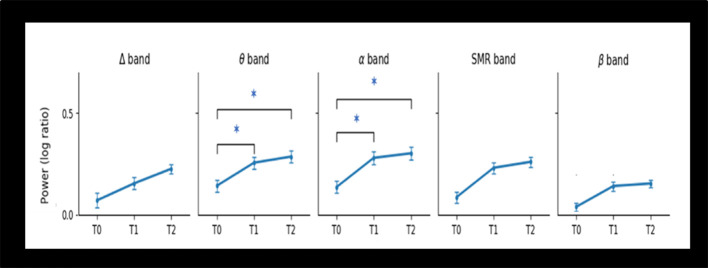
Changes in Power of EEG bands from baseline (T0), post-intervention (T1), and 1-month follow-up (T2) for the 20 elderly patients. Abbreviations: Δ, delta; θ, theta; SMR, Sensorimotor; α, alpha; β, lower beta. Note: *statistical significant difference (*p* < 0.05).

Repeated ANOVA measures reveal that log-ratio values differences were significant for EEG theta power (*F* = 4.44; *p* = 0.016) and alpha power (*F* = 3.84; *p* = 0.027) after the NF intervention, but not for the other frequency bands: delta, SMR, and beta. Paired *t*-test found that patients showed greater theta power (*p* = 0.04) and alpha power (*p* = 0.03) at T1 compared to T0, and at T2 compared to T0 (*p* = 0.03 and *p* = 0.04 respectively). However, no significant changes did occur when comparing T2 with T1 (Table 4). Figure 3 depicts the log ratio for each EEG band power at the three assessment endpoints. The graphs feature the significant increase of theta band and the alpha band following the NF training (T1) and the sustaining of these increments at a 1-month follow-up (T2).

**Table 4:** Changes on power values for each frequency band from baseline (T0) to post-NF training (T1) and 1-month follow-up (T2) for the 20 elderly patients.

The power log ratio of EEG frequency bands	T0	T1	T2				T1–T0	T2–T0	T2–T1
	(*n* = 20)	(*n* = 20)	(*n* = 20)	Repeated Measures ANOVA			Student’s *t*-test		
	mean ± SEM	mean ± SEM	mean ± SEM	*F*_(2,38)_	*p*-values	Cohen’s *f*	*p*-values		
Delta	0.22 ± 0.21	0.15 ± 0.28	0.07 ± 0.34	1.452	0.243	0.093	0.40	0.13	0.37
Theta	0.26 ± 0.24	0.23 ± 0.26	0.08 ± 0.27	4.443	0.016*	0.163	0.04*	0.03*	0.53
Alpha	0.3 ± 0.29	0.28 ± 0.30	0.14 ± 0.29	3.841	0.027*	0.212	0.03*	0.04*	0.70
SMR	0.28 ± 0.28	0.25 ± 0.29	0.14 ± 0.27	1.920	0.156	0.196	0.14	0.12	0.67
Beta	0.15 ± 0.18	0.14 ± 0.21	0.04 ± 0.2	3.052	0.059*	0.074	0.06*	0.05*	0.79

Abbreviations: SMR, sensorimotor; SEM, Standard Error Mean. Note: *Significance at *p* < 0.05.

**Figure 4:**
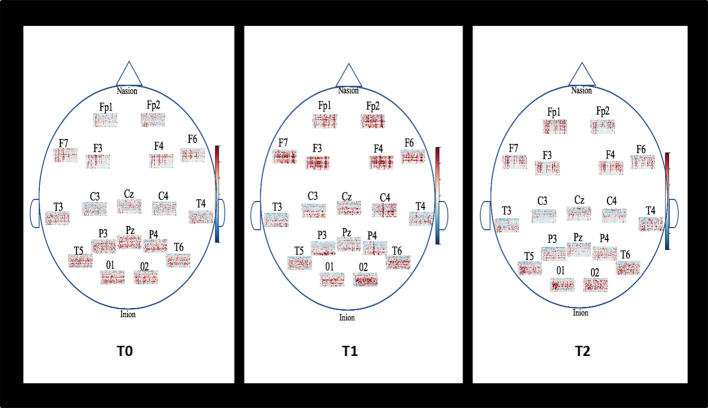
Example of topographic distribution (time-frequency) of the EEG spectral power for each electrode placed on the scalp according to the international 10-20 system for one patient. Baseline (T0), Post-NF training (T1), and 1-month follow-up (T2). The spectrograms show the estimation frequencies for each electrode at baseline, after the NF training and 1-month follow-up from one of the 20 patients. In this example, differences were observed at post-NF training with increased global activity in frontal areas (Fp1, Fp2, F3, F4, F6, F7) and central regions (C3, Cz, C4). In posterior areas, alpha activity appeared more determined than at baseline. At 1-month follow-up, the global activity got back to its previous lower global activity observed at baseline.

The spectrograms presented in Figure 4 show the estimation frequencies for each electrode at baseline, after the NF training and 1-month follow-up from one of the 20 patients. In this example, differences were observed at post-NF training with increased global activity in frontal areas (Fp1, Fp2, F3, F4, F6, F7) and central regions (C3, Cz, C4). In posterior areas, alpha activity appeared more determined than at baseline. At 1-month follow-up, the global activity got back to its previous lower global activity observed at baseline.

## Discussion

This pilot study aimed to evaluate the effects of the SMR/theta protocol NF training in older adults diagnosed with MCI. Twenty participants attended a NF training program for over 10 weeks.

### Neuropsychological and Psycho-affective Functioning in Elderly With Mild Cognitive Impairment Following Neurofeedback

We found improvements in working and episodic memory and psycho-affective dimensions of elderly patients with MCI, following 10 weeks of NF training. These findings highlight the potential benefit of NF interventions in promoting cognitive rehabilitation and preventing further impairment in this specific population. Six out of the 20 neuropsychological scores showed improvements such as the Forward digit spans that assess the working memory by processing speed and attention control. The improvements in the forward digit span persisted for another 1 month after the NF training had been discontinued. These increases in working memory and attention control can be attributed to several NF exercises within the training program, which were focused on concentrating on numbers thus stimulating these cognitive processes. Moreover, from the list of NF tasks, elderly participants expressed verbally a better interest for tasks with focused numbers.

Our results are consistent with other studies. In a randomized placebo-controlled study, Campos da Paz et al. (2018) showed that the SMR protocol could improve working memory performance and attention in healthy older adults (Campos da Paz et al., 2018). Recently, Bielas and Michalczyk (2020) demonstrated the improvement of attention capacity after a beta NF training protocol (12–22 Hz) of 20 sessions, 30 min each, with an active electrode placed at Cz in the elderly population (Bielas and Michalczyk, 2020). Jirayucharoensak et al. (2019) conducted a controlled study with amnestic MCI (aMCI) subjects treated with 20 sessions NF beta and alpha training of 30 min, two or three times a week (Jirayucharoensak et al., 2019). They used the five same NF tasks over the 20 sessions for each participant and obtained the improvement of sustained attention and spatial working memory. Our Present study that tested the SMR NF protocol in the central area of the cortex in the elderly with MCI reinforces the idea that EEG NF improves attentional processes and working memory. The compliance with the technical aspects developed by Arns et al. (2017) such as the number of sessions, the duration of the training, the trained frequency band SMR or Beta 1 at the site Cz, may be considerate as crucial to obtain effectiveness in NF training (Arns et al., 2017).

The delayed recall on RAVLT assesses the long-term consolidation process in episodic verbal memory. Scores of this variable improved significantly at postintervention but were not observed during the 1-month follow-up. Within the NF program, some situations could evolve in episodic memory functioning such as the patient’s involvement to remind task instructions at the beginning of each new session and the historical report as NF task. Patients showed strong interest and attention for this semantic support.

Jirayucharoensak et al. (2019) described their NF games, some of which were focused on calculating number but they did not link them to their results (Jirayucharoensak et al., 2019). NF training suggests being concentrate on the game without any given strategy (Jang et al., 2019). The participant is informed about the expected result but he has to find his own strategy to focus. NF principle is that the more attention is focused, the more the thresholds are reached.

A second main finding was the improvement in psycho-affective scores which reflect lower anxiety, less cognitive complaints, and better wellness of the participants. Most of the patients expressed their satisfaction with being supported.

### EEG Changes in Elderly Patients With Mild Cognitive Impairment Following EEG Neurofeedback Training

EEG studies in MCI patients usually feature a gradual shift from rapid (decreased activity in the alpha and beta bands) to slow rhythms (increased activity of delta and low theta bands; Houmani et al., 2018; Babiloni et al., 2004; Gouw et al., 2017). The present pilot study was carried out on the hypothesis that NF Training could increase rapid brain activities while reducing slow activities, and then will improve cognitive dysfunctions. We found that there were significant differences between baseline and postintervention in elderly patients in some EEG frequency bands. Indeed, alpha and theta activities improved significantly after EEG NF training. The increase in the power of the alpha, and theta bands on the resting EEGs with eyes closed, at T1 and T2, therefore, means a reinforcement of the power of these bands after the NF SMR/Theta training sessions. This reflects an improvement in brain activity. The increase in the power of theta may be debatable, but it should be emphasized that the increase in the power of the theta band may come from the fact that there is a slight decrease or a lesser increase in the power of the delta band. This means that part of the delta rhythms is mutated into theta rhythm. The brain activity is, therefore, faster after the NF training. Jurewicz et al. (2018) showed that NF training aimed at upregulating beta activity not only affected the trained beta band but also the flanking untrained alpha activity (8–12 Hz; Jurewicz et al., 2018). Their observation supports the hypothesis that activation of a frequency band may emphasize other frequency bands.

Spectrograms gave an estimation of activity for each electrode’s localization in a time dimension. In the presented example, they showed changes in EEG activities after the NF training. Electrophysiological data in AD are characterized by lower beta1 activity changes in anterior areas although normal waking EEG is usually composed of beta1 frequencies in the fronto-centrotemporal head regions with low amplitude. Working memory depends on the fronto-central regions with a correlation of a dominant beta1 activity. Thus, better beta1 oscillations are believed to maintain the current sensorimotor and cognitive state (Engel and Fries, 2010). Activity in the beta1 frequency has also traditionally been linked with motor function in central regions. It has been found that motor performance is impaired in early-stage Alzheimer’s disease but not in mild cognitive impairment (Sheridan et al., 2003; Sheridan and Hausdorff, 2007). Nevertheless, beta2 (20–30 Hz) with high amplitude can result from anxiety or a drug’s effect thus, signal interpretation has to be cautious. Alpha activity appeared more distinct in posterior regions at post-NF training than at Baseline where it was more confused among the other frequencies. Observable alpha activity in parieto-occipital regions is a sign of a normal electrophysiology functioning although, in prodromal AD state and AD, it has been well established that alpha rhythm is considerably decreased. The limitation of the spectrogram is the time window length that has to be correctly adapted to show the best resolution frequency. This can be a problem if a signal containing two components with a similar frequency which may then be received as one single component.

The results of this pilot study should be interpreted with precaution due to several limitations. Indeed, it did not include a control group such as in the double-blind, controlled, randomized study. The placebo effect could not be totally excluded. One other major limitation is the small sample size as well as the heterogeneity of cognitive profile and pathophysiology among the elderly participants.

## Conclusion

Our findings suggested that elderly subjects with cognitive dysfunctions could benefit from NF training with improvements being observed in working memory, consolidation process in memory, psycho-affective dynamics, and activity in the alpha band and theta band. Elderly patients’ motivation and concentration greatly depend on the nature of the tasks for NF training. NF training could be preferred instead of the video games considered childish by older people. Studying the learning curves during NF training by recording the amplitude values and identifying frequency peaks would allow knowing the relevance of the tasks in terms of predictor effectiveness of NF. The differential effects on EEG measures suggest that adjustments are necessary for older adults to avoid drowsiness episodes due to frequent sleep disorders in elderly people. Among the adjustments, the EEG_NF protocol has to be appropriate according to the patient’s cognitive profile and pathophysiology. We hypothesize that some patients could be more resistant than others to an SMR/theta NF protocol suggesting less potential cognitive plasticity and evolution of Alzheimer’s disease. These findings are encouraging and deserve future cognitive and neurophysiology intervention studies where brain lesions should be taken into consideration.

## Data Availability Statement

The datasets generated for this study are available on request to the corresponding author.

## Ethics Statement

The study protocol was approved by the ethics committee of the Paris Descartes University Institutional Review Board (reference code: 2017-12; 28/02/2017). All patients signed the informed consent form to participate in this study.

## Author Contributions

FM developed the study concept, wrote, and drafted the manuscript. EA and A-SR participated in implementing the trial, contributed to the draft manuscript, and helped with participant recruitment. MB, GB, and SC participated in analyzing the database, statistical data, and contributed to the draft manuscript. LD-J, Y-HW, and HL gave a critical review of the manuscript and helped with patients’ recruitment. All authors read and approved the final manuscript.

## Conflict of Interest

The project was supported by Namecheap Company, ICANN-accredited domain name registrar and web hosting company, Los Angeles, USA. Namecheap Company did not contribute to the study design or to writing the manuscript.

## Abbreviations

AD: Alzheimer’s disease
ADHD: Attention deficit hyperactivity disorder
ANOVA: Analysis of variance
CONSORT: Consolidated standards of reporting trials
K.T: Based cancellation test
EEG: electroencephalography
GDS: Geriatric depression scale
ICA: independent component analysis
MCI: Mild Cognitive Impairment
MMSE: Mini-mental state evaluation
MoCa: Montreal cognitive assessment
NF: Neurofeedback
RAVLT: Rey auditory verbal learning test
RCT: Randomized controlled trial
ROCF: Rey Osterrieth complex figure
SMR: Sensorimotor brain rhythm
WAIS-IV: Weschler adult intelligent scale fourth edition.
